# Correlations between Process Parameters and Outcome Properties of Laser-Sintered Polyamide

**DOI:** 10.3390/polym11111850

**Published:** 2019-11-09

**Authors:** Dan Ioan Stoia, Liviu Marşavina, Emanoil Linul

**Affiliations:** Department of Mechanics and Strength of Materials, Politehnica University of Timisoara, 1 Mihai Viteazu Avenue, 300 222 Timisoara, Romania; dan.stoia@upt.ro (D.I.S.); liviu.marsavina@upt.ro (L.M.)

**Keywords:** polymer processing, additive manufacturing, laser sintering, mechanical properties, sample orientation, energy density

## Abstract

As additive manufacturing (AM) becomes more accessible, correlating process parameters with geometric and mechanical properties is an important topic. Because the number of process variables in AM is large, extensive studies must be conducted in order to underline every particular influence. The study focuses on two variables—part orientation in the orthogonal horizontal plane and energy density—and targets two outcomes—geometric and tensile properties of the parts. The AM process was conducted on selective laser sintering (SLS) machine EOS Formiga P100 using EOS white powder polyamide (PA2200). After finishing the sinterization process, the parts were postprocessed, measured, weighted, and mechanically tested. The geometric evaluation and mass measurements of every sample allowed us to compute the density of all parts according to the sinterization energy and orientation, and to determine the relative error of every dimension. By conducting the tensile testing, the elastic and strength properties were determined according to process variables. A linear trend regarding sample density and energy density was identified. Also, large relative dimensional errors were recorded for the lowest energy density. Mechanical properties encountered the highest value for the highest energy density at a 45° orientation angle.

## 1. Introduction

Polymers are a highly diverse class of materials that possess unique properties, which make them useful in a wide variety of applications [1,2,3]. Furthermore, the engineering applications of high-strength polymeric materials and their lightweight composites are endless and still increasing significantly due to their low cost and ease of manufacture [4,5,6]. The geometrical versatility of additive manufacturing (AM) [7,8,9], together with the availability of the market, makes the technology increasingly popular for industry and home users [10,11,12]. Many authors studied this research direction, but inconsistencies in part properties remain a concern [13,14,15].

Liao et al. [16] conducted extensive studies to determine the porosity and elastic properties in both the static and dynamic conditions of extruded polylactic acid (PLA) filaments. High porosity values were recorded for the bottom layers of the samples, while lower values of 1–2% was determined for the top layers. Also, a linear relation between porosity and elastic modulus was proposed. Another laser processing of polyamide (PA6) is welding. The influence of pulsed laser on the mechanical characteristics of welded PA6 joints was studied by Pereira et al. [17]. Mechanical tests were carried out for welded joint, evidencing 55% of based material strength. A correlation between the 3D porosity and the elastic properties of PLA samples manufactured by Fused Deposition Modeling (FDM) was established by Wang et al. [18]. Here, prediction diagrams of Young modulus and Poisson’s ratio were plotted according to pore size distribution. The samples formed very similar pore size distribution regardless of the process parameters used, and lower porosity led to better mechanical properties. A study on fresh and recycled PA12 powder manufactured at different orientations was conducted by Feng et al. [19] using an FDM machine. They determined the mechanical properties in tensile and bending, as well as also the impact strength, of the PA12 powder. The best properties were associated with X-direction and fresh powder, while samples manufactured using recycled powder conducting to a property decrease of 10–15%.

Hesse et al. [20] proposed an interesting study on electrostatic interaction during powder spreading using PA2200 and EOS Formiga machines for the sintering process. The results revealed higher voltage peaks for the aged material than for the fresh materials. This finding can be correlated with different densities obtained when using fresh or recycled polyamide powder. The characterization of sintered polyamide was studied by Pilipović et al. [21] and Dizon et al. [22]. Their work underlined some mechanical properties according to the energy density used during the manufacturing process. Also, the orientation of the parts in the building chamber was taken into consideration. The fracture properties of PLA samples were determined according to part orientation by Ahmed and Susmel [23], while metallic materials produced by laser melting were studied by Razavi and Berto [24] and Solberg et al. [25] and on ceramics by Arab et al. [26]. In their work, the fracture toughness was determined using a significant number of samples. Using a mechanical mixture of PA, aluminum, PA2200, a comparison of mechanical properties according to the orientation in XY plane was found by Stoia et al. [27]. Three different orientations were considered for both materials but the same energy density was considered for all samples.

Beside mechanical properties, geometrical characteristics of additive manufactured parts are also related to orientation, energy density, and other variables. The geometric measurements and stereomicroscopy of the structure were used by some authors in order to characterize AM parts [28,29,30]. Some authors focused their work on the geometrical characterization of samples obtained at different orientations and/or energy density by measuring the size and shape and by inspecting the surface of the parts using stereomicroscopy, without taking into accounts the mechanical properties aspects. The inconsistency in dimensions and properties of selective laser sintering (SLS) polymers was approached by Goodridge et al. [31], who underlined the effect of uneven temperature distribution in the building volume, together with other process variables: orientation, energy, layer thickness, and powder condition.

This paper presented investigations on mechanical and geometrical parameters, taking into account two process variables: Energy density and orientation angle. The statistical validation of data and the correlations of the process parameters with the outcome properties were determined by means of ANOVA analysis and Pearson’s coefficient.

## 2. Materials and Methods

### 2.1. Materials

One powder material, polyamide PA2200, was used in the study. Polyamide PA2200 was produced by Electro Optical Systems-EOS GmbH, Krailling, Germany.

EOS white powder polyamide (PA2200) is a versatile material based on polyamide PA12 which, in laser sinterization form, exhibits good chemical, physical, and mechanical properties despite its porosity. The properties are directly influenced by the additive process. A set of parameters determined by the manufacturer in its own conditions can be found in datasheet: Grain size according to ISO 13320-11 is 56 µm (average) [32]; bulk density according to EN ISO 60 is 0.45 g/cm³ [33]; melting point according to EN ISO 11357–1 is 172–180 °C [34]; Vicat softening temperature according to EN ISO 306 is 163 °C for B/50 method and 181 °C for A/50 method [35].

Important properties of PA2200 are connected with human body interaction. According to EN ISO 10993–1 [36] and USP/level VI/121 °C, it manifests biocompatibility so it can be used within the human body. In compliance with the EU Plastics Directive 2002/72/EC, it can also be used for food contact [37].

### 2.2. Methods

The AM process was conducted on an EOS Formiga P100 machine (EOS GmbH Electro Optical Systems, Krailling, Germany) using polyamide PA2200 powder. Three different energy densities (ED) and five orientation angles (OA) were considered. The tensile testing was conducted on an INSTRON 8800 testing machine (Instron, MA, USA).

#### 2.2.1. Process Parameter Setup

**Figure 1:**
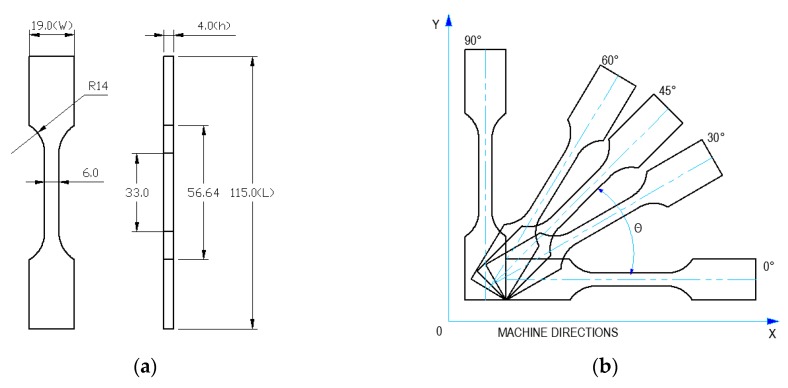
Sample model: Shape and size (**a**); orientation angles (OA) of the samples in XY building plane (**b**).

The fabrication process started with 3D modelling of the sample using SolidWorks 2013 and in accordance to the geometrical reference of ISO 527–1:2012 (Figure 1a) [38]. In every machine job, all considered OA (0°, 30°, 45°, 60°, and 90°) had to be manufactured. Thus, the parts were virtually positioned in the machine’s frame using Magics 12 (Materialise, Leuven, Belgium) (Figure 1b). For each OA at each ED, six samples were manufactured, resulting a total number of 75 samples for this study. The safety distances between all surfaces of neighboring samples were dx, dy = 6 mm. This distance prevents powder hardening caused by high local heat. Because there was no room for all samples in one building plane, offset layer groups were created using a growing safety distance of dy = 15 mm.

**Figure 2:**
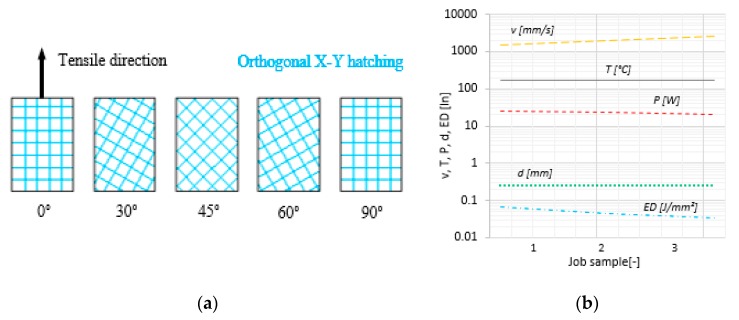
OA grid and controllable parameters: Hatching lines (**a**) and P (power), v (velocity), d (scan spacing), T (temperature), and ED (energy density) graphic relation (**b**).

The association between the orientation of the sample and the orthogonal trajectories of the laser beam during the hatching process led to a grid-like structure inside of the sintered volumes. The theoretical structure resulted from combining alternating orthogonal trajectories of the laser beam, and the OA is presented in the Figure 2a.

The following step involved the division of the volume in consecutive layers of 0.1 mm, which was offset using EOS RP Tools application (EOS GmbH Electro Optical Systems, Krailling, Germany). In this stage, the laser trajectories were generated according to the shape of the sample. An error checking was performed to eliminate the sharp edges and other geometries that were impossible to be generated.

Using EOS PSW software (EOS GmbH Electro Optical Systems, Krailling, Germany), the job file was further prepared. All geometries were scaled up using the scaling factors provided in Table 1, and assigned values of the process parameters are also presented. The parameter abbreviation signifies: P—laser power; v—scanning velocity; ED—energy density; d—scan spacing; h—beam offset; T—temperature of the building chamber; T_r_—temperature of the removal chamber; t—layer thickness; SF—scaling factors on XY plane.

The laser beam power (P), scanning velocity (v), and scan spacing (d) were the three parameters that built up the ED required for sinterization, according to the Equation (1) [39,40]. In this study, only the velocity and power were considered variables, while the scan spacing was constant. The additional energy required for sinterization was provided by the heating system of the machine. The dependencies of the controllable process parameters (P, v, T, d, ED) are presented in the Figure 2b.

(1) $$\mathrm{ED}\;[J/{\mathrm{mm}}^{2}]=\frac{P\;[W]}{v\;[\mathrm{mm}/s]\cdot d\;[\mathrm{mm}]}$$

#### 2.2.2. Formiga P100 Machine Setup

The EOS Formiga P100 (EOS GmbH Electro Optical Systems, Krailling, Germany) is a 30W CO_2_ laser machine designed for layer-by-layer sinterization of plastic powders. The powder is deposited in two barrels from which the ducting system of the machine delivers the powder on the building plane using a sweeping blade. The two main chambers of the machine are the building chamber and removal chamber. These chambers are physically separated by a steel plate, making it possible to set up different temperatures within the chambers (163–170 °C for the building chamber and 150–153 °C for the removal chamber). The heat is generated by three electrical sources. From there, radiation the heat is transferred by convection to the powder, rising its temperature. The powder heating ensures softening on one hand and reduction of the temperature gradient on Z direction on the other hand. Beside temperature, the oxygen content is controlled by the nitrogen generator.

The laser beam exits the laser tube and passes through a series of rotating mirrors up to the optic chamber. Here, deflecting mirrors allow the deviation of the beam according to the geometrical information of the part. The laser spot diameter is Φ = 0.42 mm and represents a physical parameter of the machine. It unequivocally influences the minimum wall thickness that can be achieved using this type of machine. Also, the ED modifies the curing zone of the laser spot, with values of Φ = 0.68 mm recorded by [41]. This further influences the minimum wall thickness that can be obtained.

After the building process, which took around 8 h to finish each ED, the parts were cooled down for 16 h. The samples were then removed from the machine and cleaned out using an air blasting equipment.

#### 2.2.3. Measurements and Data Processing

In the next stage of the study, all samples were measured along X, Y, and Z directions using a Mitutoyo digital caliper of 0.02 mm accuracy. All measurements were repeated four times in order to obtain an average value for every dimensional parameter. The linear parameters measured were: Total length of the sample (L) and width of the sample at ends (W), both corresponding to the XY plane, and thickness of the sample (H), corresponding to the Z direction.

In order to compute the density, square samples of 19 mm × 19 mm × 4 mm were cut off from tensile samples. One square sample was cut off for every OA at each ED. The squares were measured again and weighted using a Kern laboratory balance of 0.01 g accuracy. Using their mass and volume, the density was determined.

The statistical significance of measured and determined parameters was checked using one-way analysis of variance (ANOVA). The correlation between the technological parameters used in manufacturing process and the outcome investigated parameter were evidenced using Pearson’s correlation.

#### 2.2.4. Tensile Tests

**Figure 3:**
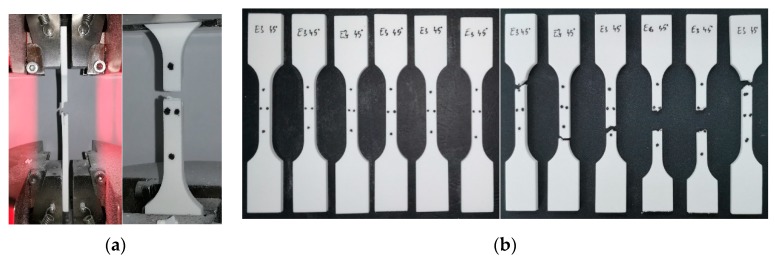
The fixed sample in the tensile testing grips (**a**) and samples before/after the tensile test (**b**).

The quasi-static tensile tests were conducted on a 25 kN INSTRON 8800 testing machine (Instron, MA, USA) at room temperature. All samples were numbered, and four dots were painted in the middle section to allow use in a video extensometer (see Figure 3a). All tests were run up to the failure point using a 5 mm/min loading speed. Force, displacement, longitudinal, and transversal strains were recorded during tests for further data processing [42].

For each orientation angle and each energy density, six dog-bone samples were tested according to ISO 527 standard [38]. For the highest accuracy of the results, only the closest five measurements were taken into consideration, with the sixth being excluded. Figure 3b shows the samples before and after the tensile test.

## 3. Results and Discussion

In the following subsections, the geometric and tensile properties are graphically presented, together with the detailed conditions in which the results were obtained.

### 3.1. Geometric Properties

The geometric measurements were acquired four times for every linear parameter for all samples. At the end of the measurements, an averaged value of length (L), width (W), and height (H) for every sample according to OA and ED was recorded. Taking into account the nominal dimension of the sample and the real dimensions, the relative percentage error was computed using Equation (2). Here, $v_{M}$ represents measured values, while $v_{N}$ represents the nominal value defined in design phase (L = 119 mm, W = 19 mm, and H = 4mm).

(2) $$\mathrm{Err}\;\left[\%\right]=\left|\frac{v_{M}-v_{N}}{v_{N}}\right|\times 100$$

**Figure 4:**
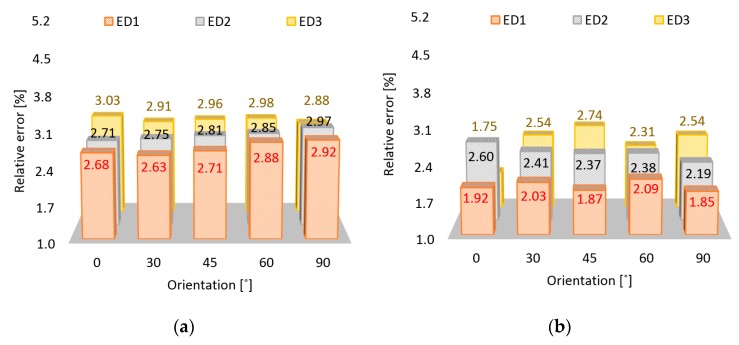
Relative error of the sample size according to OA and energy density (ED): Length (**a**); width (**b**).

**Figure 5:**
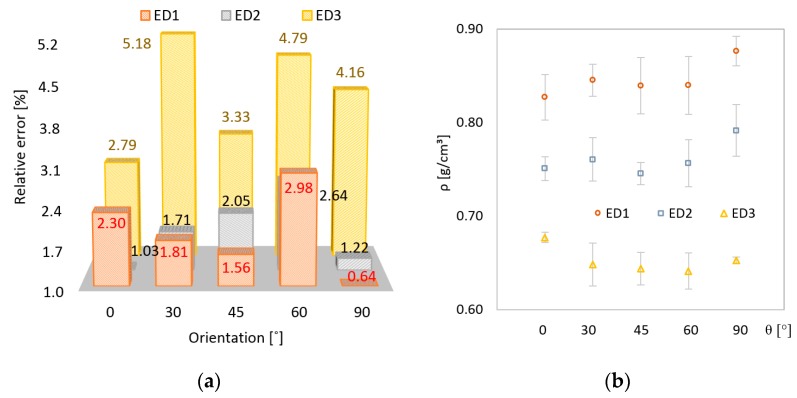
Relative error of the sample size and density, according to OA and ED: Thickness (**a**); density (**b**).

In the Figure 4 and Figure 5a, the relative error of length, width, and thickness are presented using the same vertical scale. The chart blocks were arranged to depict all five orientation angles, while the color of the blocks was associated with an energy density. As an overall observation, no sample experienced zero error in L, W, or H dimensions. A uniform distribution of error almost independent on ED and OA values was determined for length (Figure 4a). It appeared that the larger the dimension, the less influenced the dimension was by the two process parameters.

For the second dimension, W, smaller errors were determined. The maximum deviation from the nominal value was recorded for ED3 and 45° OA. A 10% to 20% error decrease was observed when the ED increased from the lowest value used in the study to the highest value (Figure 4b) Higher energy led to better particle bonding, proving to also influence the dimensional stability in a convenient manner.

Significant changes in dimensional error were recorded for thickness (Figure 5a), which belongs to the growing direction of the samples (Z direction). Errors as high as 5% relating to nominal size were recorded for ED3. This large spread of data for thickness can be placed on heat transfer through the volume of the powder. At the same time, less energy density led to a poor arrangement of powder particles. In the Z direction, the dimension was also influenced by the compression exerted by the top layers of the powder on the bottom layer.

From the angular orientation point of view, no clear tendency of the dimensional error can be identified.

The density computed according to ED and OA proved the influence of the two parameters on the structure of the samples. Average density values with standard deviations are presented in the Figure 5b. Higher values of density were recorded for higher energy densities at a relatively equal data spread. The highest density values can be found in 90° OA. The single reasonable explanation of this behavior is the orientation of the part in relation to the direction of powder spreading. The sweeping blade may generate a local settle down of the powder.

Table 2 presents the analysis of variance for total length of the sample (L), taking into account the OA and ED. This parameter was selected for statistical testing due to the relatively uniform error observed in the Figure 4a in order to prove whether L was significantly influenced by the ED. The sum of squares was presented for both within the groups (WG) and between the groups (BG), as well as the *p*-value [43]. The results showed that ED significantly influenced the total length in all OA, with P values much lower than 0.05.

The linear dependence of the density with ED is statistically proven in the Table 3. The analysis of variance evidenced significant differences in densities (*p*-value << 0.05) obtained using different ED for all orientation angles.

### 3.2. Tensile Properties

This section presents the results from tensile tests performed on dog-bone samples. These experimental results are essential for designing AM parts and can be used as a baseline.

**Figure 6:**
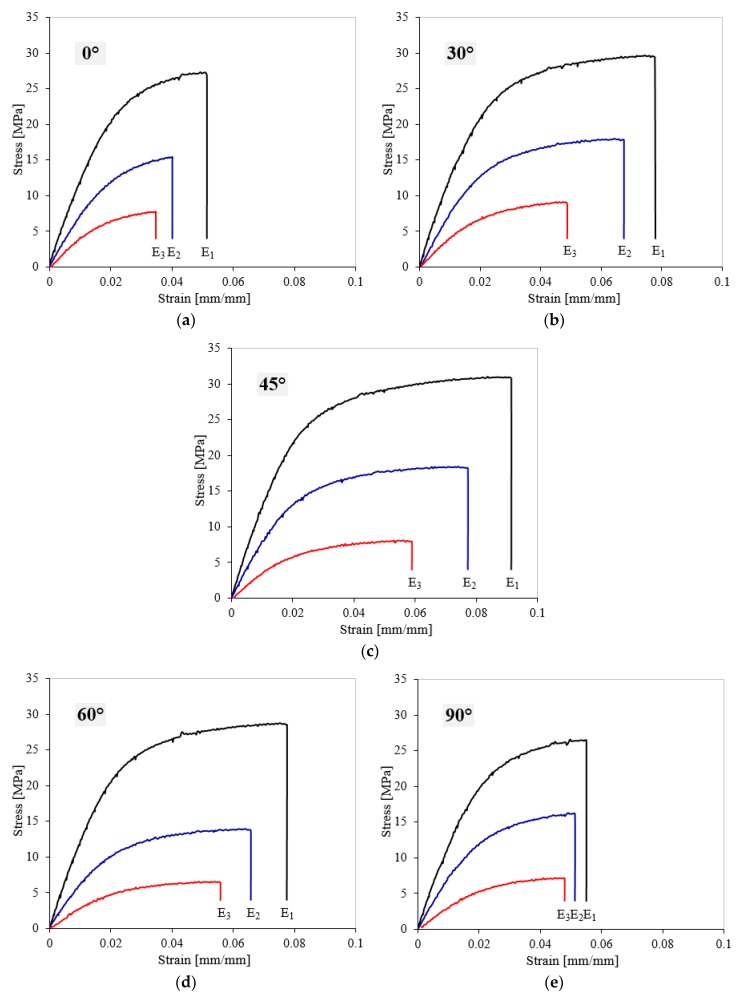
Tensile stress–strain curves of 3D-laser-sintered PA2200 for different energy densities and orientations: (**a**) 0°; (**b**) 30°; (**c**) 45°; (**d**) 60°, and (**e**) 90°.

Figure 6 presents the stress–strain curves of the investigated PA2200 polyamide for different energy densities (ED1, ED2, ED3) and different sample orientation angles (0°, 30°, 45°, 60°, 90°).

These curves clearly indicate that there was a significant modification in both stiffness and strength as a function of the ED and OA. All curves showed a linear-elastic behavior followed by a smooth hardening until fracture [44,45]. From the experimental tensile stress–strain curves, it can be seen that the yield point could not be specifically determined. The failure collapse mechanism was a quasi-brittle one, however, under tensile loading conditions, small plastic deformation for all samples were observed. Furthermore, for all five orientations, the highest stress–strain behavior was obtained for ED1, followed by ED2 and ED3.

Figure 6 highlights the important effect of energy density on the tensile behavior of the 3D-printed PA2200 polyamide. In order to quantify this effect, the main tensile properties (Young’s modulus, E; quasi-elastic limit, σ_e_; yield strength, σ_y_; tensile strength, σ_m_; elongation at break, ε_b_ and energy absorption at break, W_b_) were studied as a function of the sample orientation angle [46]. All these mechanical properties were determined using ISO 527 standard [38] and are listed in Table 4, Table 5 and Table 6 for all tested samples, according to ED and OA.

**Figure 7:**
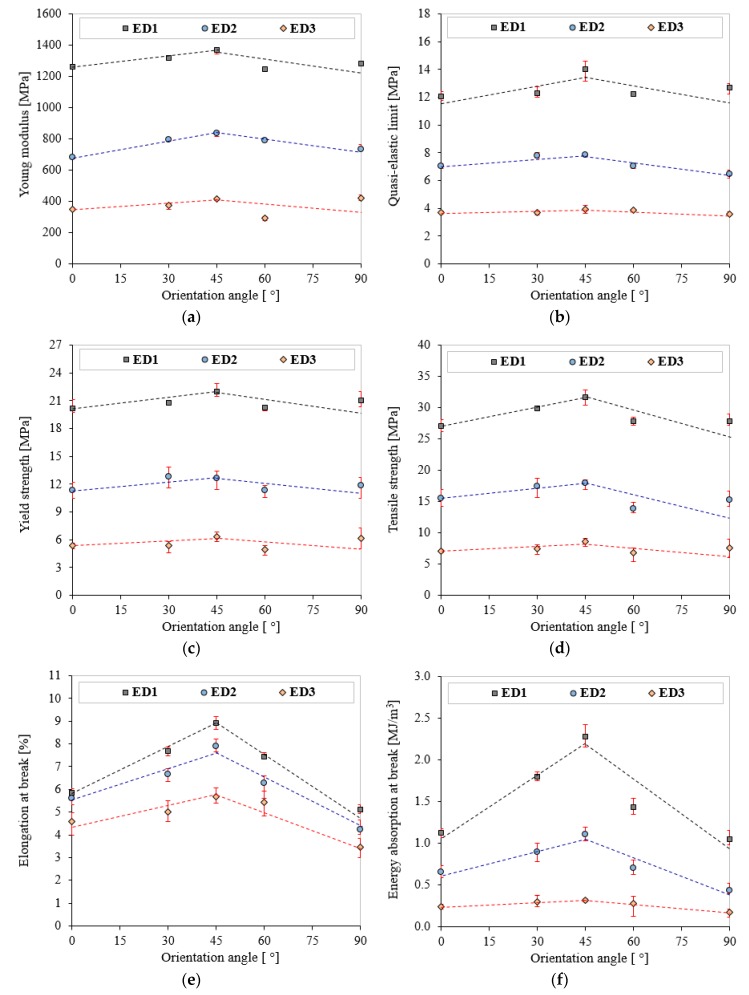
Tensile mechanical properties of laser-sintered PA2200 according to ED (ED1, ED2, and ED3) and OA (0°, 30°, 45°, 60°, and 90°): Young modulus (**a**); quasi-elastic limit (**b**); yield strength (**c**); tensile strength (**d**); elongation at break (**e**); energy absorption at break (**f**).

For an easier visualization and interpretation of the results, Figure 7 shows the variation of the tensile properties according to ED and OA. The data reported in all graphs are averages over the five tests, together with their standard deviations [47]. From Figure 7, it can be easily observed that the ED played fundamental role in the tensile behavior of laser-sintered PA2200 polyamide. Thereby, an increase of up to 623.86% of the absorption energy at break was obtained if an energy density ED1 of 0.067 J/mm^2^ was used compared to ED3 of 0.034 J/mm^2^. The smallest increase (which can still represent an advantage depending on the field of use of the parts) of only 57.24% was the elongation at break. The elastic (E, σ_e_) and strength (σ_y_, σ_m_) properties also increased considerably by up to 250%. Therefore, choosing the adequate ED is a very important aspect for obtaining good laser sintering parts [48]. The mechanisms explaining the tensile properties dependence on ED are the different temperatures developed in the process. Higher sinterization energy leads to larger bridges between the polymer particles. This increases the part’s density, and subsequently, to the increase of its mechanical properties [49,50,51].

On the other hand, for all three energy densities, it was observed that the high properties were obtained for an orientation of 45°. The percentage increase of the mechanical properties for the orientation of 45° varied from 9.5% for Young’s modulus to 153.5% for W_b_. At 45°, the orthogonal trajectories of the laser beam, in combination with the orientation of the part in XY plane, generated an oblique sinterization hatching with respect to the direction of tensile (Figure 2a). This relation between sinterization and tensile direction may prevent the crack growth when the part is subjected to mechanical loading. Also, the σ_y_ and σ_m_ properties increased by up to 28.8% in the case of the samples printed at an OA of 45°. Contrariwise, no significant data differences concerning the tensile properties between the pairs of 0°/90° and 30°/60° orientation angles were observed. Therefore, the results concerning the changes in the mechanical properties as a function of OA are quite different to those for the ED.

For statistical validation of the findings, the tensile strength was analyzed using one-way ANOVA (Table 7), taking into consideration all energies at all orientations. Statistically significant differences between tensile strength at ED1, ED2, and ED3 were determined for all OA, *p*-value << 0.05.

Pearson’s correlations are expressed in Table 8, presenting the linear relation between the outcome variables L, ρ, and σ_m_ and technological parameters ED and OA. A positive linear correlation very close to 1 was observed for all three outcome parameters and the energy density. Tensile strength showed poor correlation with OA, regardless of the ED value. The length of the samples had the highest correlated parameter with OA, with a negative linear relation identified for ED1 and ED2.

## 4. Conclusions

This paper investigated the geometric and tensile properties of selective laser-sintered PA2200 polyamide, considering different orientation angles (OA) and energy densities (ED). For this purpose, three different ED (ED1, ED2, and ED3) and five OA (0°, 30°, 45°, 60°, and 90°) were considered.

The following conclusions can be drawn:

- The relative dimensional error for length (L), width (W), and thickness (H) ranged from 0.64% to 5.18%, with the larger error recorded for the lowest energy density (ED3).
- The largest variability was obtained for (H), measured along Z axis.
- The parameters L, ρ, and σ_m_ manifested positive linear correlations of 0.928, 0.974, and 0.998 with ED, respectively.
- Poor correlation with OA was determined for ρ and σ_m._
- The most promising tensile properties were obtained for ED1 at OA of 45°.
- One-way ANOVA analysis for L, ρ, and σ_m_ parameters showed statistically significant differences for ED1, ED2, and ED3 at every OA (*p*-value << 0.05).

## Figures and Tables

**Table 1:** Laser sintering parameters for all samples.

Code	P [W]	v [mm/s]	ED [J/mm^2^]	d [mm]	h [mm]	T [°C]	T_r_ [°C]	t [mm]	SF [%]
E1	25	1500	0.067		0.15				
E2	23	2000	0.046	0.25		169.5	159	0.1	2.3
E3	21	2500	0.034						

**Table 2:** ANOVA analysis of total length.

Parameter		Orientation Angle									
		0°		30°		45°		60°		90°	
ED		SS	*p*-value	SS	*p*-value	SS	*p*-value	SS	*p*-value	SS	*p*-value
	BG	0.5170	8 × 10^−5^	0.2737	1 × 10^−4^	0.2072	1 × 10^−4^	0.0618	4 × 10^−3^	0.0282	1 × 10^−2^
	WG	0.1362		0.0775		0.0598		0.0417		0.0276	

**Table 3:** ANOVA analysis of density.

Parameter		Orientation Angle									
		0°		30°		45°		60°		90°	
ED		SS	*p*-value	SS	*p*-value	SS	*p*-value	SS	*p*-value	SS	*p*-value
	BG	0.0337	8 × 10^−5^	0.0585	8 × 10^−5^	0.0574	9 × 10^−5^	0.0596	2 × 10^×4^	0.0764	1 × 10^−5^
	WG	0.0015		0.0027		0.0027		0.0039		0.0020	

**Table 4:** Tensile properties of SLS PA2200 polyamide for ED1.

Orientation Angle α [°]	Young’s Modulus E [MPa]	Quasi-elastic Limit σ_e_ [MPa]	Yield Strength σ_c_ [MPa]	Tensile Strength σ_m_ [MPa]	Elongation at Break ε_b_ [%]	Energy Absorption W_b_ [MJ/m^3^]
0	1253.9	12.21	19.86	26.70	5.59	1.15
	1271.4	11.79	19.70	26.21	5.86	1.06
	1276.1	12.11	20.45	27.73	5.98	1.18
	1266.8	12.43	21.10	28.14	5.87	1.14
	1252.5	11.89	19.84	26.54	5.96	1.08
30	1222.8	12.37	19.66	27.32	7.28	1.55
	1266.2	12.82	21.03	28.98	7.46	1.70
	1264.8	12.59	20.63	28.75	7.77	1.77
	1229.5	11.95	19.79	26.58	6.24	1.05
	1263.4	11.37	19.93	27.69	8.48	1.11
45	1375.3	14.07	22.33	32.16	8.93	2.38
	1364.8	14.00	22.19	32.25	9.07	2.35
	1370.5	13.82	21.74	30.98	8.79	2.18
	1356.7	13.91	21.74	31.32	8.94	2.25
	1374.3	14.18	22.07	31.54	8.79	2.22
60	1222.8	12.37	19.66	27.32	7.28	1.55
	1266.2	12.82	21.03	28.98	7.46	1.70
	1264.8	12.59	20.63	28.75	7.77	1.77
	1229.5	11.95	19.79	26.58	6.24	1.05
	1263.4	11.37	19.93	27.69	8.48	1.11
90	1273.7	12.31	21.76	28.71	5.34	1.15
	1282.2	12.26	20.65	27.14	5.25	1.05
	1276.7	13.01	20.62	27.18	4.96	0.98
	1291.3	12.89	21.96	28.98	4.94	1.03
	1278.7	12.96	20.32	27.35	5.13	1.02

**Table 5:** Tensile properties of SLS PA2200 polyamide for ED2.

Orientation Angle α [°]	Young’s Modulus E [MPa]	Quasi-elastic Limit σ_e_ [MPa]	Yield Strength σ_c_ [MPa]	Tensile Strength σ_m_ [MPa]	Elongation at Break ε_b_ [%]	Energy Absorption W_b_ [MJ/m^3^]
0	674.98	7.21	10.43	14.29	5.76	0.59
	693.32	6.86	12.23	16.91	5.48	0.73
	674.39	7.07	10.9	15.13	5.71	0.67
	674.15	6.84	10.92	14.93	5.47	0.65
	692.49	7.16	12.03	16.38	5.53	0.64
30	804.77	7.73	12.83	16.79	6.76	0.93
	792.98	8.04	13.81	18.41	6.77	1.00
	784.55	7.66	12.76	18.21	6.46	0.87
	797.62	7.61	13.11	17.06	6.89	0.92
	799.66	7.89	11.61	16.82	6.35	0.77
45	836.94	7.68	12.88	18.04	8.21	1.20
	849.08	7.92	13.18	19.18	8.10	1.08
	837.14	8.01	13.15	17.90	7.72	1.12
	836.20	7.75	12.04	18.24	7.26	1.03
	826.66	7.82	11.89	16.11	8.22	1.10
60	781.20	7.11	11.79	14.26	6.22	0.62
	800.66	7.08	12.14	13.83	6.45	0.70
	806.64	7.03	10.11	13.95	6.02	0.71
	771.67	6.92	11.82	14.51	6.56	0.69
	800.60	6.94	10.75	12.94	6.04	0.78
90	736.41	6.49	12.07	15.45	4.01	0.42
	721.53	6.73	10.46	14.33	4.15	0.52
	737.56	6.38	11.33	14.19	4.01	0.41
	722.98	6.52	12.55	15.77	4.43	0.44
	763.34	6.19	12.68	16.68	4.64	0.40

**Table 6:** Tensile properties of SLS PA2200 polyamide for ED3.

Orientation Angle α [°]	Young’s Modulus E [MPa]	Quasi-elastic Limit σ_e_ [MPa]	Yield Strength σ_c_ [MPa]	Tensile Strength σ_m_ [MPa]	Elongation at Break ε_b_ [%]	Energy Absorption W_b_ [MJ/m^3^]
0	347.09	3.58	5.30	7.29	4.98	0.26
	350.12	3.79	5.01	6.86	3.97	0.26
	349.14	3.67	5.33	7.19	4.81	0.24
	350.57	3.76	5.42	7.22	4.25	0.21
	344.46	3.65	5.60	6.82	4.88	0.23
30	358.74	3.76	4.82	7.04	4.88	0.31
	380.32	3.78	4.76	5.98	5.49	0.37
	370.57	3.54	5.61	7.88	4.60	0.24
	381.04	3.77	5.73	8.05	4.76	0.29
	383.39	3.62	5.80	8.14	5.29	0.29
45	428.82	3.98	6.69	9.17	5.70	0.29
	413.28	3.91	6.82	9.28	3.85	0.16
	428.56	3.93	6.30	8.40	6.35	0.37
	419.73	3.95	6.36	8.59	6.50	0.40
	393.71	3.98	5.60	7.77	5.91	0.35
60	294.71	3.85	5.41	7.28	5.22	0.27
	296.92	3.93	5.16	7.17	5.85	0.31
	296.65	4.11	5.03	6.96	5.35	0.27
	281.21	3.56	4.35	6.01	5.18	0.29
	294.73	3.94	4.79	6.58	5.58	0.27
90	405.36	3.61	6.29	7.98	3.84	0.20
	441.70	3.52	6.47	7.77	3.46	0.18
	418.94	3.49	5.04	6.02	3.01	0.11
	412.91	3.53	5.45	6.77	3.51	0.15
	436.97	3.75	7.30	9.03	3.49	0.21

**Table 7:** ANOVA analysis of tensile strength.

Parameter		Orientation Angle									
		0°		30°		45°		60°		90°	
ED		SS	*p*-value	SS	*p*-value	SS	*p*-value	SS	*p*-value	SS	*p*-value
	BG	1006.7	2 × 10^−13^	1045.2	6 × 10^-13^	1340.3	3 × 10^−14^	1148.5	3 × 10^−14^	1055.4	3 × 10^−12^
	WG	7.6139		9.8336		7.6750		6.5014		12.9031	

**Table 8:** Pearson’s correlation for outcome—technological parameters.

Outcome Parameter	Technological Parameters			
	Energy Density (ED)	Orientation Angle		
		OA (at ED1)	OA (at ED1)	OA (at ED1)
Length (L)	0.9285	−0.7640	−0.8412	0.4529
Density (ρ)	0.9744	0.5480	0.5021	−0.4415
Tensile strength (σ_m_)	0.9988	0.1307	−0.2569	0.0730
